# Cytotoxic Labdane Diterpenes from *Hedychium ellipticum* Buch.-Ham. ex Sm.

**DOI:** 10.3390/molecules21060749

**Published:** 2016-06-09

**Authors:** Sineenard Songsri, Nuchnipa Nuntawong

**Affiliations:** Department of Chemistry and Center for Innovation in Chemistry, Faculty of Science, Chiang Mai University, Chiang Mai 50200, Thailand; n_boxtree@hotmail.com

**Keywords:** *Hedychium ellipticum*, labdane-type diterpene, cytotoxic activity, antimycobacterial activity

## Abstract

In order to reveal the constituents and their biological activities, we carried out a phytochemical study on *Hedychium ellipticum* Buch.-Ham. ex Sm. (Zingiberaceae). Ten labdane diterpenoids (**1**–**10**) were isolated from the rhizomes of *H. ellipticum* for the first time. Their structures were identified on the basis of spectroscopic analyses including two-dimensional NMR and comparison with literature data. All of these compounds were evaluated for their antimycobacterial activity against *Mycobacterium tuberculosis* and cytotoxicity against KB, MCF7, NCI-H187 and Vero cells. The result showed that compounds **1** and **7** exhibited moderate activity against *Mycobacterium tuberculosis* and compounds **4**, **6** and **7** displayed remarkable cytotoxic activity. This is the first report on the presence of all compounds in *H. ellipticum* and the first time that their structure activity relationship has been discussed.

## 1. Introduction

Several species of *Hedychium* are used widely in traditional medicines for treatment of skin diseases, stomach ailments, cough and apoplexy, and as an analgesic, anti-inflammatory, antimicrobial and anti-rheumatic treatment [1,2]. Previous phytochemical studies on *Hedychium* species revealed the presence of diarylheptanoids, sesquiterpenes and diterpenes, some of which showed hepatoprotective effects [3], antiinflammatory activities [4] and cytotoxicity against various cancer cells [5,6,7,8,9,10,11,12]. *Hedychium* *ellipticum* Buch.-Ham. ex Sm. (Zingiberaceae), cultivation from Doi Suthep overlooking Chiang Mai in Thailand has bright green rolled bracts. No detailed phytochemical and pharmacological investigation on *H. ellipticum* has been reported, except for a report on the chemical composition of leave and rhizome essential oils, which showed that (*E*)- nerolidol and 1,8-cineole were the major constituents [13,14]. The methanolic extracts of rhizomes showed potent activity to inhibit the biosynthesis of leukotriene B_4_ in bovine polymorphonuclear leukocytes and the essential oils exhibited moderate-to-good Fe^2+^ chelating activity [15]. In our preliminary investigation on the bioactivities of *Hedychium* plants, we found that the crude *n*-hexane and CH_2_Cl_2_ extracts of *H.* *ellipticum* rhizomes showed moderated antimycobacterial activity against *Mycobacterium* *tuberculosis* and cytotoxic activity against human cancer cell lines. Therefore, the aim of the present work is to determine the chemical constituents from the rhizomes of *H. ellipticum* and to evaluate their antimycobacterial activity against *Mycobacterium tuberculosis* and cytotoxicity against KB, MCF7, NCI-H187 and Vero cells. The structure–activity relationships of all isolated compounds were also reported.

## 2. Results

**Figure 1:**
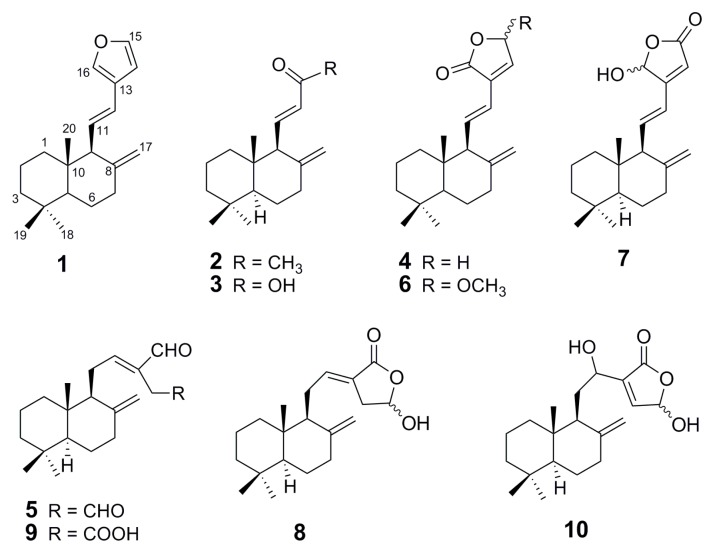
The structures of compounds **1**–**10** from *H. ellipticum*.

Phytochemical investigation of the rhizomes of *H. ellipticum* resulted in the isolation of 10 labdane-type diterpenes: coronarin E (**1**) [10], (*E*)-15,16-bisnorlabda-8(17),11-dien-13-one (**2**) [16], (*E*)-14,15,16-trinorlabda-8(17),11-dien-13-oic acid (**3**) [3], villosin (**4**) [10], (*E*)-labda-8(17),12-dien-15,16-dial (**5**) [3], 15-methoxylabda-8(17),11,13-trien-15,16-olide (**6**) [17], 16-hydroxylabda-8(17),11,13-trien-15,16-olide (**7**) [17], coronarin D (**8**) [8], zerumin A (**9**) and zerumin B (**10**) [18] (Figure 1). Their structures were determined by spectroscopic methods and by comparison of their spectral data with those reported in the literature. These compounds are known but they were isolated from this plant for the first time. This phytochemical investigation of *H. ellipticum* has shown that its chemistry differs from other species of *Hedychium*. Compounds **1**, **4**, **5**, **6** and **8** were isolated from the rhizomes of *Hedychium coronarium* J. Koenig [3,4,5,8,12], *Hedychium villosum* Wall. [1] and *Hedychium gardnerianum* Sheppard ex Ker Gawl. [10]. Interestingly, compounds **2**, **3**, **7**, **9** and **10** have not been reported from any species of the *Hedychium* and could be regarded as an important chemotaxanomic marker. All isolated compounds were evaluated for their antimycobacterial activity against *Mycobacterium tuberculosis* and cytotoxicity against three human tumor cell lines, human oral cavity cancer cell (KB), human breast cancer cell (MCF7) and human small cell lung cancer cell (NCI-H18) and non-cancerous (Vero) cell lines. The results of the antimycobacterial activity against *Mycobacterium tuberculosis* of all compounds are given in Table 1. Compounds **7** and **1** exhibited moderate activity against *Mycobacterium* *tuberculosis* with MIC 6.25 and 12.5 μg/mL, respectively, while compounds **2**, **3**–**6**, and **8**–**10** were inactive. All compounds exhibited over 20-fold inferior than standard drug streptomycin. To the best of our knowledge, this is the first report for the antimycobacterial activity of all compounds. In a comparison of all compounds, furan ring or α,β-unsaturated γ-lactone ring is essential for the antimycobacterial activity. Change in an α,β-unsaturated γ-lactone ring did affect the activity as indicated in the case of compounds **4**, **6**, **7** and **10**. The hydroxyl group at C-16 significantly enhanced the activity of compound **7**, which suggested that the relative hydrophilicity of this part of the molecule contributes to the activity. However, lack in activity of compound **10**, implying that the orientation of cyclic hemiacetal α,β-unsaturated γ-lactone is also influenced the activity. Although the structure–activity relationships of these compounds were not conclusively determined, this finding provided a useful starting point for antimycobacterial activity of labdan-type diterpenoids.

In previous studies, some labdane diterpenes from the species of *Hedychium* were found to show cytotoxicity. Therefore, compounds **1**–**10** were also evaluated for cytotoxic activities against three cancer cell lines: human oral cavity cancer cell (KB), human breast cancer cell (MCF7) and human small cell lung cancer cell (NCI-H18) (Table 1). All of the 10 labdane-type diterpenoids exhibited generally stronger cytotoxic effect against NCI-H187 cell than those against KB and MCF-7 cells. Results revealed that compounds **4**, **6** and **7** possessed potent cytotoxic activity against the NCI-H187 cell with an IC_50_ value of 0.12, 0.90 and 0.72 μg/mL, respectively. In addition, compound **4** showed higher cytotoxicity than a positive control, ellipticine (IC_50_ 0.44 μg/mL) which agree with previous reported [10]. Its toxicity was undetectable at concentration less than 50 μg/mL and very selective for NCI-H187 cell. Furthermore, compound **7** was also possessed potent cytotoxic activity against the KB cell and moderate activity against the MCF7 cell with an IC_50_ value of with an IC_50_ value of 0.92 and 2.89 μg/mL, respectively. However, compound **7** was very toxic to Vero cells (IC_50_ 5.37 μg/mL) and not selective for tested cell lines. Compounds **2**, **3**, **5**, **8** and **9** showed weak to no effect, while **1** and **10** showed moderate to weak effect on all tested cell lines. It should be noted that compound **1** has been reported to exhibit moderate cytotoxicity against P388, B16 and SNU-1 cells with IC_50_ 10.0, 18.8 and 8.0 μg/mL [19] but weak cytotoxicity against NCI-H187 cell with IC_50_ 49.73 μM [10]. Compounds **5** and **8** have been reported to exhibit cytotoxic activity against V-79 cell with IC_50_ 18.5 and 17.0 μg/mL, respectively [5,12]. Compound **8** exhibited weaker cytotoxic activity against KB (40.01 μg/mL) and NCI-H187 (25.72 μg/mL) cells than previous reported which showed moderate activities with IC_50_ 14.7 and 9.8 μg/mL [8]. However, it has been reported to displayed potent cytotoxic activity against A-549 cell line with the LC_50_ value of 14.0 μM and weak cytotoxicity against SK-N-SH, MCF-7 and HeLa cells with LC_50_ 32.0, 21.9 and 60.6 μM, respectively [12]. To the best of our knowledge, this is the first report for the cytotoxic activities of compounds **2**, **3**, **6**, **7**, **9** and **10**. On comparing the cytotoxic activity of compounds **1**–**10**, drastic decrease or lack in cytotoxicity of compounds **1**–**3**, **5**, **8** and **9** against tested cell lines, implying that an α,β-unsaturated γ-lactone ring plays a crucial role in mediating cytotoxic activity. Furthermore, change in the lactone moiety did affect the cytotoxicity. In a comparison of compound **4** with **6** and **10**, the hydroxyl or methoxyl substitution at C-15 significantly reduced the cytotoxicity against all the tested cells. Nevertheless, among the compounds **7** and **10**, the orientation of cyclic hemiacetal α,β-unsaturated γ-lactone also influenced the cytotoxicity against all the tested cells. This is similar to their cytotoxicities against K562 cancer cell lines which was reported previously [6]. It is noteworthy to mention that the hydroxyl group at C-16 significantly enhanced the cytotoxicity of compound **7** against KB and MCF-7 cell lines, which suggested that the relative hydrophilicity of this part of the molecule contributes to the cytotoxicity. However, the lack of *trans* double bond conjugated with the lactone ring seemed to decrease cytotoxic activity, as indicated in the case of compound **10**.

## 3. Materials and Methods

### 3.1. General Experimental Procedures

Thin layer chromatography (TLC): precoated silica gel 60 *F*_254_ plates (0.2 mm thick; Merck). The TLC spots were visualized under UV light and by heating the plates after spraying with anisaldehyde-H_2_SO_4_ reagent. Column chromatography (CC): SiO_2_ (finer than 0.063 mm; Merck), *Sephadex LH-20* (Merck). Melting points: Electrothermal apparatus, uncorrected. Optical rotations: JASCO-1020 polarimeter. IR spectra: Tensor 27 FT-IR Spectrophotometer; KBr pellets. ^1^H- and ^13^C-NMR spectra: Bruker AVANCE 400 NMR spectrometer (400 and 100 MHz, resp.). HREIMS: Q-TOF 2™ mass spectrometer with a Z-spray™ ES source (Micromass, Manchester, UK). EI-MS: Agilent-HP 5973 mass spectrometer.

### 3.2. Plant Material

The rhizomes of *H. ellipticum* were collected from Chiang Mai province of Thailand, in January 2007. A voucher specimen (06-609 J.F. Maxwell) was deposited with the CMU Herbarium, Department of Biology, Faculty of Science, Chiang Mai University, Thailand.

### 3.3. Extraction and Isolation

Air-dried powdered rhizome (920 g) of *H.* *ellipticum* was successively extracted with *n*-hexane and CH_2_Cl_2_ (4 L × 3) at room temperature. The solvents were combined and evaporated to dryness under reduced pressure to yield a brownish residue of *n*-hexane (44.26 g) and CH_2_Cl_2_ (39.50 g) extracts. The *n*-hexane (44.26 g) extract was subjected to CC (*n*-hexane/CH_2_Cl_2_ 100:1 to 0:1, gradient) to furnish five fractions, *Frs. H1*–*H*5. *Fr. H1* (5.15 g) was successively subjected to CC (*n*-hexane) to afford compound **1** (2.84 g). *Fr. H2* (4.18 g) was successively subjected to CC (*n*-hexane/CH_2_Cl_2_ 100:1) to yield four fractions, *Frs. H2.1*–*H2.4*. *Fr. H2.4* (1.6 g) was further separated by CC (*n*-hexane/EtOAc 98:2) to give five fractions, *H2.4a*–*H2.4e*. *Fr.* *H2.4e* was further purified by CC (*n*-hexane/EtOAc 98:2) to afford compound **2** (35.8 mg). *Fr. H4* (8.60 g) was successively subjected to CC (CH_2_Cl_2_) to yield four fractions, *Frs. H4.1*–*H4.4*. *Fr. H4.4* (3.8 g) was further separated by CC (CH_2_Cl_2_/MeOH 98:2) to give three fractions, *H4.4a*–*H4.4c*. *Fr.* *H4.4b* (1.1 mg) was further purified by CC (*n*-hexane/EtOAc 98:2) to afford compound **3** (20.1 mg). The CH_2_Cl_2_ extract (39.50 g) was subjected to a quick column (SiO_2_ finer than 0.045 mm *n*-hexane/CH_2_Cl_2_ 100:0 to 0:100, CH_2_Cl_2_/EtOAc 95:5 to 0:100), EtOAc/MeOH 95:5 to 50:50, gradient) to yield five fractions, *Frs. C1*–*C5*. *Fr. C3* (7.85 g) was further separated by CC (*n*-hexane/CH_2_Cl_2_ 60:40) to give three subfractions, *C3.1*–*C3.3*. *Fr. C3.2* (2.9 g) was further purified by CC (*n*-hexane/CH_2_Cl_2_ 60:40) to give three subfractions, *Frs. C3.2a*–*C3.2c*. Compound **4** (216.2 mg) was crystallized directly with CH_2_Cl_2_/*n*-hexane from *Fr. C3.2c* (1.8 g). *Fr. C3.3* (1.2 g) was further separated by CC (CH_2_Cl_2_ 100%) to give three fractions, *Frs. C3.3a*–*C3.3c*. *Fr.* *C3.3b* (225.4 mg) was further purified by CC (*n*-hexane/EtOAc 90:10) to afford compound **5** (45.9 mg). *Fr. C4* (16.42 g) was further separated by CC (CH_2_Cl_2_/MeOH 97:3) to give *Frs. C4.1* and *C4.2*. *Fr. C4.1* (6.8 g) was rechromatographed by CC (*n*-hexane/EtOAc 80:20) to give three subfractions, *Frs. C4.1a*–*C4.1c*. *Fr. C4.1a* (2.1 g) was further purified by CC (CH_2_Cl_2_/MeOH 97:3) to give four subfractions, *Frs. C4.1a1*–*C4.1a4*. *Fr. C4.1a3* (0.9 g) was further purified by CC (*n*-hexane/EtOAc 80:20) to afford compounds **6** (35.8 mg) and **7** (57.8 mg). *Fr. C4.1a4* (0.7 g) was further separated by CC (*n*-hexane/EtOAc 70:30) to afford compounds **8** (23.8 mg) and **9** (11.3 mg). *Fr. C4.2* (5.6 g) was separated by CC (CH_2_Cl_2_/MeOH 97:3) to give three fractions, *C4.2a*–*C4.2c*. *Fr.* *C4.2b* (1.4 g) was further purified by CC (*Sephadex LH-20*; MeOH) to afford compound **10** (41.7 mg). All compounds **1**–**10** were identified by interpretation of their spectral data including EIMS, ^1^H- and ^13^C-NMR (including DEPT135, COSY, HMQC and HMBC experiments), as well as by comparison of their spectral data with those reported in the literature. (NMR data of all compounds are given in the Supplementary Materials).

### 3.4. Antimycobacterial Activity

The antimycobacterial activity was assessed against *Mycobacterium tuberculosis* H_37_Ra using the green fluorescent protein microplate assay (GFPMA) [20]. The lowest drug concentration effecting and inhibition of ≥90% was considered the MIC. The standard drugs, rifampicine, streptomycin, isoniazid, oflaxacin and ethambutol showed MIC values of 0.00312–0.025, 0.156–0.313, 0.0234–0.0468, 0.391–0.781 and 0.234–0.469 μg/mL, respectively.

### 3.5. Cytotoxic Activity

The cytotoxicity against human tumor cells was determined by resazurin microplate assay (REMA) which was a modified method of the use of a fluorescent dye for mammalian cell cytotoxicity [21]. The cytotoxicity against non-cancerous cells, African green monkey kidney (Vero) cell line was determined by the green fluorescent protein (GFP) detection [22]. Briefly, cells at a logarithmic growth phase were harvested and diluted to 9 × 10^4^ cells/mL in fresh medium. Successively, 5 μL of test sample, diluted in 10% DMSO and 45 μL of cells suspension were added to 384-well plates. Plates were incubated at 37 °C in 5% CO_2_ incubator for 5 days. After that, 12.5 μL of 62.5 μg/mL resazurin solution was added to each well and the plates were then incubated at 37 °C for 4 h. Fluorescence signal was measured using SpectraMax M5 multidetection microplate reader at dual wavelengths of 530 and 590 nm. For non-cancerous cells such as the African green monkey kidney (Vero) cell line, the cytotoxicity was determined by the green fluorescent protein (GFP) detection [22]. Briefly, cells at a logarithmic growth phase were harvested and diluted to 3.3 × 10^4^ cells/mL in fresh medium. Successively, 5 μL of test sample, diluted in 0.5% DMSO and 45 μL of cells suspension were added to 384-well plates. Plates were incubated at 37 °C in 5% CO_2_ incubator for 4 days. Fluorescence signals were measured on day zero, for subsequent background subtraction, and on day 4 using SpectraMax M5 multi-detection microplate reader in the bottom-reading mode with excitation and emission wavelengths of 485 and 535 nm. IC_50_ values were derived from dose-response curves, using six concentrations of 2-fold serially diluted samples, by the SOFTMax Pro software (Molecular device). The standard drugs ellipticine exhibited IC_50_ values against KB, NCI-H187 and Vero cell at 0.302, 0.440 and 1.345 μg/mL and doxorubicin exhibited IC_50_ values against MCF7 at 0.663 μg/mL. The selectivity index corresponded to the IC_50_ value determined for cytotoxicity of isolated compounds on Vero cells divided by the IC_50_ determined for cancer cells.

## 4. Conclusions

We showed a low antimycobacterial activity and demonstrated the cytotoxic activities of some compounds among those isolated. The cytotoxicity results provided baseline information for the possible use of villosin (**4**), 15-methoxylabda-8(17),11,13-trien-15,16-olide (**6**) and 16-hydroxylabda-8(17),11,13-trien-15,16-olide (**7**) for the development of antitumor drugs. All compounds were identified from this plant for the first time and compounds (*E*)-15,16-bisnorlabda-8(17),11-dien-13-one (**2**), (*E*)-14,15,16-trinorlabda-8(17),11-dien-13-oic acid (**3**), 16-hydroxylabda-8(17),11,13-trien-15,16-olide (**7**), zerumin A (**9**) and B (**10**) have not been isolated from any species of *Hedychium*. The isolation and identification of 10 compounds provide the first significant phytochemical report for this plant species and may be used as a foundation for further chemotaxonomic studies. The resulting information would be of some significance for drug discovery and development research and for further studies on the genus.

## Figures and Tables

**Table 1:** Anti- *Mycobacterium tuberculosis* H37Ra (Anti-TB) and cytotoxic activities of isolated compounds.

Compounds	Anti-TB	Cytotoxicity (IC_50_) in μg/mL (μM)				Selectivity Index		
	(MIC, μg/mL)	KB	MCF7	NCI-H187	Vero Cell	KB	MCF7	NCI-H187
Coronarin E (**1**)	12.5	9.67 (34.00)	Inactive	18.06 (63.50)	25.37 (89.20)	2.6	0.5	1.4
(*E*)-15,16-Bisnorlabda-8(17),11-dien-13-one (**2**)	Inactive	36.99 (142.05)	49.22 (189.02)	21.67 (83.22)	Undetectable ^2^	1.4	1.0	2.3
(*E*)-14,15,16-Trinorlabda-8(17),11-dien-13-oic acid (**3**)	Inactive	Inactive	Inactive	49.23 (187.64)	Undetectable ^2^	-	-	1.0
Villosin (**4**)	Inactive	4.1 (13.65)	8.5 (28.29)	0.12 (0.40)	Undetectable ^2^	12.2	5.9	>416.7
(*E*)-Labda-8(17),12-dien-15,16-dial (**5**)	Inactive	29.05 (96.05)	22.55 (74.56)	11.17 (36.93)	22.52 (74.46)	0.8	1.0	2.0
15-Methoxylabda-8(17),11,13-trien-15,16-olide (**6**)	Inactive	23.82 (72.08)	49.16 (148.77)	0.9 (2.72)	45.65 (138.14)	1.9	0.9	50.7
16-Hydroxylabda-8(17),11,13-trien-15,16-olide (**7**)	6.25	0.91 (2.75)	2.89 (8.75)	0.72 (2.18)	5.37 (16.25)	5.9	1.9	7.5
Coronarin D (**8**)	Inactive	40.01 (125.64)	16.25 (51.03)	25.72 (80.77)	48.89 (153.53)	1.2	3.0	1.9
Zerumin A (**9**)	Inactive	47.41 (148.88)	Inactive	33.46 (105.07)	49.48 (155.38)	1.0	1.0	1.5
Zerumin B (**10**)	Inactive	13.13 (39.26)	13.28 (39.71)	6.14 (18.36)	24.69 (73.82)	1.9	1.9	4.0
Ellipticine ^1^	-	0.302 (1.23)	-	0.44 (1.79)	1.345 (5.46)	4.5	-	3.1
Doxorubicin ^1^	-	0.117 (0.22)	0.663 (1.22)	0.053 (0.10)	-	-	-	-

^1^ Standard drug; ^2^ Undetectable at concentration ≤50 μg/mL (or 192.01, 190.57 and 166.43 μM for compounds **2**, **3** and **4**, respectively).

## Supplementary Materials

Supplementary materials can be accessed at: http://www.mdpi.com/1420-3049/21/6/749/s1.
