# Supplementary material for: Cytotoxic Labdane Diterpenes from Hedychium ellipticum Buch.-Ham. ex Sm
Source: Molecules. 2016 Jun 9;21(6):749. doi: 10.3390/molecules21060749 (PMC6273991; doi:10.3390/molecules21060749)
Supplement: Supplementary file 1 [file molecules-21-00749-s001.pdf]

Supplementary Materials: Cytotoxic Labdane Diterpenes from *Hedychium ellipticum* Buch.-Ham. ex Sm.

Sineenard Songsri, Apichart Suksamrarn and Nuchnipa Nuntawong

Spectroscopic Data of Compounds 1–10

Coronarin E (**1**): pale yellow oil; $[\alpha]_D^{29}$ = +19.70 (*c* 0.57, $CHCl_3$); EIMS: *m/z* 284 $[M]^+$ (100); 269 (9), 147 (95), 137 (28), 69 (14), 55 (20), 41(17); ^{1}H-NMR (400 MHz, $CDCl_3$): δ 0.82 (3H, s, H-20), 0.83 (3H, s, H-19), 0.88 (3H, s, H-18), 1.01 (dt, *J* = 14.4, 4.2 Hz, H-1α), 1.11 (1H, dd, *J* = 12.5, 2.6 Hz, H-5), 1.18 (1H, m, H-3α), 1.35 (1H, m, H-6α), 1.40 (1H, m, H-3β), 1.42 (1H, m, H-2α), 1.47 (1H, m, H-1β), 1.50 (1H, m, H-2β), 1.70 (1H, m, H-6β), 2.10 (1H, dt, *J* = 13.2, 5.2 Hz, H-7β), 2.37 (1H, d, *J* = 9.8 Hz, H-9), 2.45 (1H, m, 7β), 4.51 (1H, d, *J* = 1.6 Hz, H-17a), 4.74 (1H, d, *J* = 1.6 Hz, H-17b), 5.96 (1H, dd, *J* = 15.7, 9.8 Hz, H-11), 6.19 (1H, d, *J* = 15.7 Hz, H-12), 6.52 (1H, s, H-14), 7.33 (2H, s, H-15, H-16); ^{13}C-NMR (100 MHz, $CDCl_3$): δ 15.00 (C-20), 19.12 (C-2), 21.95 (C-19), 23.39 (C-6), 33.56 (C-4, 18), 36.77 (C-7), 39.15 (C-10), 40.78 (C-1), 42.32 (C-3), 54.63 (C-5), 61.48 (C-9), 107.65 (C-14), 107.96 (C-17), 121.75 (C-12), 124.51 (C-13), 128.29 (C-11), 139.59 (C-16), 143.24 (C-15), 150.22 (C-8); FTIR (neat): ν = 2922, 1643, 1156 cm^{-1}.

(E)-15,16-Bisnorlabda-8(17),11-dien-13-one (**2**): pale yellow solid; mp 146.0–147.0 °C; $[\alpha]_D^{29}$ = −8.50 (*c* 0.67, $CHCl_3$); EIMS: *m/z* 260 $[M]^+$ (47) ; 245 (16), 217 (35), 137 (60), 81 (92), 69 (30), 43(58); ^{1}H-NMR (400 MHz, $CDCl_3$): δ 0.84 (3H, s, H-19), 0.89 (2 × 3H, s, H-18, H-20), 1.05 (1H, m, H-1α), 1.10 (1H, dd, *J* = 12.5, 2.6 Hz, H-5), 1.20 (1H, m, H-3α), 1.37 (1H, m, H-1β), 1.42 (1H, m, H-2α), 1.39 (1H, m, H-6α), 1.44 (1H, m, H-3β), 1.54 (1H, m, H-2β), 1.71 (1H, m, H-6β), 2.09 (1H, dt, *J* = 12.5, 4.8 Hz, H-7α), 2.48 (1H, d, *J* = 10.0 Hz, H-9), 2.45 (1H, overlapping, H-7β), 4.40 (1H, br s, H-17a), 4.79 (1H, br s, H-17b), 6.07 (1H, d, *J* = 15.8 Hz, H-12), 6.87 (1H, dd, *J* = 15.8, 10.0 Hz, H-11); ^{13}C-NMR (100 MHz, $CDCl_3$): δ 15.11 (C-20), 18.99 (C-2), 21.92 (C-19), 23.23 (C-6), 33.54 (C-4), 33.57 (C-18), 36.61 (C-7), 39.33 (C-10), 40.86 (C-1), 42.09 (C-3), 54.45 (C-5), 60.79 (C-9), 108.61 (C-17), 133.57 (C-12), 146.72 (C-11), 148.61 (C-8), 198.18 (C-13); FTIR (neat): ν = 2936, 1664, 1258, 898 cm^{-1}.

(E)-14,15,16-Trinorlabda-8(17),11-dien-13-oic acid (**3**): white amorphous; $[\alpha]_D^{29}$ = +6.44 (*c* 0.34, $CHCl_3$); HREIMS: $[M + Na]^+$ 285.1830 (calcd for $C_{17}H_{26}O_2Na$, 285.1831); ^{1}H-NMR (400 MHz, $CDCl_3$): δ 0.84 (3H, s, H-19), 0.89 (2 × 3H, s, H-18, H-20), 1.03 (1H, m, H-1α), 1.09 (1H, dd, *J* = 12.5, 2.6 Hz, H-5), 1.20 (1H, m, H-3α), 1.37 (1H, m, H-1β), 1.40 (1H, m, H-2α), 1.39 (1H, m, H-6α), 1.44 (1H, m, H-3β), 1.54 (1H, m, H-2β), 1.71 (1H, m, H-6β), 2.08 (1H, dt, *J* = 13.2, 5.0 Hz, H-7α), 2.51 (1H, d, *J* = 10.5 Hz, H-9), 2.45 (1H, overlapping, H-7β), 4.42 (1H, br s, H-17a), 4.79 (1H, br s, H-17b), 5.83 (1H, d, *J* = 15.5 Hz, H-12), 7.15 (1H, dd, *J* = 15.5, 10.5 Hz, H-11); ^{13}C-NMR (100 MHz, $CDCl_3$): δ 14.99 (C-20), 18.99 (C-2), 21.91 (C-19), 23.22 (C-6), 33.54 (C-4), 33.56 (C-18), 36.61 (C-7), 39.32 (C-10), 40.78 (C-1), 42.12 (C-3), 54.45 (C-5), 108.83 (C-17), 123.06 (C-12), 148.25 (C-9), 150.88 (C-11), 148.25 (C-8), 171.48 (C-13); FTIR (neat): ν = 3449, 2934, 1684, 1460, 1201, 737 cm^{-1}.

Villosin (**4**): white amorphous powder; mp 124.5–125.0 °C; $[\alpha]_D^{29}$ = +3.94 (*c* 0.17, $CHCl_3$); EIMS: *m/z* 300 $[M]^+$ (19) ; 285 (10), 257 (4), 189 (6), 137 (100), 123 (22), 55 (15), 41 (20); ^{1}H-NMR (400 MHz, $CDCl_3$): δ 0.80 (3H, s, H-20), 0.87 (3H, s, H-19), 0.89 (3H, s, H-18), 1.00 (1H, m, H-1α), 1.09 (1H, dd, *J* = 12.5, 2.5 Hz, H-5), 1.17 (1H, m, H-3α), 1.35 (1H, m, H-3β), 1.38 (1H, m, H-6β), 1.41 (1H, m, H-1β), 1.46 (1H, m, H-2α), 1.51 (1H, m, H-2β), 1.70 (1H, m, H-6α), 2.08 (1H, dt, *J* = 13.4, 5.1 Hz, H-7α), 2.37 (1H, d, *J* = 10.1 Hz, H-9), 2.44 (1H, ddd, *J* = 13.4, 4.0, 2.0 Hz, H-7β), 4.50 (1H, br s, H-17a), 4.76 (1H, br s, H-17b), 4.81 (2H, br s, H-15), 6.11 (1H, d, *J* = 15.8 Hz, H-12), 6.90 (1H, dd, *J* = 15.8, 10.1 Hz, H-11), 7.15 (1H, br s, H-14); ^{13}C-NMR (100 MHz, $CDCl_3$): δ 15.14 (C-20), 19.19 (C-2), 22.03 (C-19), 23.45 (C-6), 33.66 (C-4, 18), 36.83 (C-7), 39.31 (C-10), 40.89 (C-1), 42.29 (C-3), 54.75 (C-5), 62.28 (C-9), 69.69 (C-15), 108.49 (C-17), 120.75

(C-12), 129.56 (C-13), 136.92 (C-11), 142.55 (C-14), 149.50 (C-8), 172.46 (C-16); FTIR (neat): ν = 2925, 1754, 1640, 1086, 1052, 947, 902, 833 cm^{-1}.

(E)-Labda-8(17),12-dien-15,16-dial (**5**): pale yellow oil; $[\alpha]_D^{29}$ = +8.61 (*c* 0.26, $CHCl_3$); EIMS: *m/z* 302 $[M]^+$ (31); 273 (8), 177 (13), 137 (100), 81 (85); ^{1}H-NMR (400 MHz, $CDCl_3$): δ 0.73 (3H, s, H-20), 0.82 (3H, s, H-19), 0.89 (3H, s, H-18), 1.09 (1H, m, H-1α), 1.15 (1H, dd, *J* = 12.4, 2.1 Hz, H-5), 1.19 (1H, m, H-3α), 1.35 (1H, m, H-6α), 1.42 (1H, m, H-3β),1.53 (1H, m, H-2α), 1.58 (1H, m, H-2β), 1.70 (1H, m, H-1β), 1.75 (1H, m, H-6β), 1.90 (1H, d, *J* = 10.8 Hz, H-9), 2.03 (1H, dt, *J* = 12.9, 4.8 Hz, H-7α), 2.33 (1H, m, H-11a), 2.41 (1H, m, H-7β), 2.50 (1H, ddd, *J* = 16.8, 6.1, 2.7 Hz, H-11b), 3.39/3.46 (2H, AB quatet, *J* = 16.8 Hz, H-14), 4.36 (1H, br s, H-17a), 4.86 (1H, br s, H-17b), 6.77 (1H, t, *J* = 6.5 Hz, H-12), 9.63 (2H, br s, H-15); ^{13}C-NMR (100 MHz, $CDCl_3$): δ 14.44 (C-20), 19.27 (C-2), 21.73 (C-19), 24.10 (C-6), 24.68 (C-11), 33.58 (C-4, 18), 37.84 (C-7), 39.35 (C-14), 39.60 (C-10), 39.23 (C-1), 41.96 (C-3), 55.37 (C-5), 56.53 (C-9), 107.87 (C-17), 134.84 (C-13), 148.03 (C-8), 160.05 (C-12), 193.63 (C-16), 197.38 (C-15); FTIR (neat): ν = 2925, 1694, 1647, 1419, 1282, 891 cm^{-1}.

15-Methoxylabda-8(17),11,13-trien-15,16-olide (**6**): yellow oil; $[\alpha]_D^{29}$ = +10.36 (*c* 0.52, $CHCl_3$); HREIMS: $[M + H]^+$ 331.2279 (calcd for $C_{21}H_{31}O_3$, 331.2273); ^{1}H-NMR (400 MHz, $CDCl_3$): δ 0.84 (3H, s, H-19), 0.87 (3H, s, H-20), 0.89 (3H, s, H-18), 0.99 (1H, dt, *J* = 13.1, 2.6 Hz, H-1α), 1.08 (1H, dd, *J* = 12.5, 2.3 Hz, H-5), 1.18 (1H, dt, *J* = 13.3, 3.5 Hz, H-3α), 1.39 (1H, m, H-3β), 1.37 (1H, m, H-6α), 1.41 (1H, m, H-2α), 1.47 (1H, m, H-1β), 1.52 (1H, m, H-2β), 1.71 (1H, m, H-6β), 2.07 (1H, dt, *J* = 13.1, 5.0 Hz, H-7α), 2.37 (1H, d, *J* = 10.1 Hz, H-9), 2.43 (1H, obscured, H-7β), 4.47 (1H, br s, H-17a), 4.76 (1H, br s, H-17b), 5.76 (2H, br s, H-15), 6.08 (1H, d, *J* = 15.8 Hz, H-12), 6.79 (1H, br s, H-14), 6.95/6.97 (1H, dd, *J* = 15.5, 10.1 Hz, H-11); ^{13}C-NMR (100 MHz, $CDCl_3$): δ 15.07 (C-20), 19.07 (C-2), 21.93 (C-19), 23.32 (C-6), 33.57 (C-4, 18), 36.71 (C-7), 39.34 (C-10), 40.82 (C-1), 42.24 (C-3), 54.66 (C-5), 56.83/56.91 (OCH_3), 62.25/62.26 (C-9), 108.49/108.53 (C-17), 120.27/120.29 (C-12), 132.90 (C-13), 139.55 (C-11), 139.27 (C-14), 149.18 (C-8), 169.62 (C-16); FTIR (neat): ν = 3442, 2926, 1766, 1460, 937 cm^{-1}.

16-Hydroxylabda-8(17),11,13-trien-15,16-olide (**7**): pale yellow oil; $[\alpha]_D^{29}$ = +24.09 (*c* 0.63, $CHCl_3$); HREIMS: $[M + Na]^+$ 339.1938 (calcd for $C_{20}H_{28}O_3Na$, 339.1936); ^{1}H-NMR (400 MHz, $CDCl_3$): δ 0.84 (3H, s, H-19), 0.86 (3H, s, H-20), 0.89 (3H, s, H-18), 1.02 (1H, m, H-1α), 1.09 (1H, dd, *J* = 12.6, 2.3 Hz, H-5), 1.20 (1H, m, H-3α), 1.38 (1H, m, H-1β), 1.40 (1H, m, H-6α), 1.41 (1H, m, H-2α), 1.43 (1H, m, H-3β), 1.54 (1H, m, H-2β), 1.71 (1H, m, H-6β), 2.10 (1H, m, H-7α), 2.44 (1H, m, H-7β), 2.47 (1H, d, *J* = 10.6 Hz, H-9), 4.38/4.48 (1H, br s, H-17a), 4.78 (1H, br s, H-17b), 5.85 (1H, br s, H-14), 6.27/6/29 (2H, s, H-16), 6.31 (1H, d, *J* = 16.0 Hz, H-12), 6.59/6.62 (1H, dd, *J* = 16.0, 10.4 Hz, H-11); ^{13}C-NMR (100 MHz, $CDCl_3$): δ 15.09/15.16 (C-20), 19.02/19.07 (C-2), 21.93 (C-19), 23.22 (C-6), 33.55 (C-4), 33.58 (C-18), 36.59/36.62 (C-7), 39.50/39/61 (C-10), 40.84/40.99 (C-1), 42.14 (C-3), 54.51/54.53 (C-5), 62.16/62.11 (C-9), 98.00/98.02 (C-16), 108.53/108.97 (C-17), 115.33 (C-14), 120.72/122.78 (C-12), 144.05/144.13 (C-11), 148.61/148.90 (C-8), 161.52 (C-13), 172.03 (C-15); FTIR (neat): ν = 3373, 2927, 1747, 1643, 1129, 891 cm^{-1}.

Coronarin D (**8**): pale yellow oil; [$[\alpha]_D^{29}$ = +13.25 (*c* 0.85, $CHCl_3$); HREIMS: $[M + Na]^+$ 341.2090 (calcd for $C_{20}H_{30}O_3Na$, 341.2093); ^{1}H-NMR (400 MHz, $CDCl_3$): δ 0.72 (3H, s, H-20), 0.81 (3H, s, H-19), 0.88 (3H, s, H-18), 1.07 (1H, m, H-1α), 1.12 (1H, dd, *J* = 12.6, 3.0 Hz, H-5), 1.20 (1H, m, H-3α), 1.33 (1H, m, H-6β), 1.41 (1H, m, H-3β), 1.52 (1H, m, H-2α), 1.58 (1H, m, H-2β), 1.69 (1H, m, H-1β), 1.75 (1H, m, H-6α), 1.99 (1H, dt, *J* = 12.9, 4.6 Hz, H-7α), 2.21 (1H, m, H-11a), 2.35 (1H, m, H-11b), 2.37 (1H, m, H-7β), 2.71 (1H, br d, *J* = 16.6 Hz, H-14a), 2.86 (1H, obscured, H-9), 3.03 (1H, dd, *J* = 15.5, 3.4 Hz, H-14b), 4.35/4.40 (1H, s, H-17a), 4.83/4.81 (1H, s, H-17b), 5.94 (2H, dd, *J* = 3.3, 2.3 Hz, H-15), 6.76 (1H, m, H-12); ^{13}C-NMR (100 MHz, $CDCl_3$): δ 14.36 (C-20), 19.33 (C-2), 21.73 (C-19), 24.10 (C-6), 24.54 (C-11), 35.57 (C-4, 18), 33.58 (C-14), 37.79 (C-7), 39.46 (C-10), 39.26 (C-1), 42.00 (C-3), 55.33 (C-5), 56.16 (C-9), 95.96 (C-15), 107.35/107.63 (C-17), 124.17 (C-13), 143.74/143.66 (C-12), 147.93/148.14 (C-8), 170.50 (C-16); FTIR (neat): ν = 3381, 2938, 1737, 1458, 1177, 943 cm^{-1}.

Zerumin A (**9**): yellow oil; $[\alpha]_D^{29}$ = +12.76 (*c* 0.59, $CHCl_3$); HREIMS: $[M + Na]^+$ 341.2095 (calcd for $C_{20}H_{30}O_3Na$, 341.2093); ^{1}H-NMR (400 MHz, $CDCl_3$): δ 0.74 (3H, s, H-20), 0.84 (3H, s, H-19), 0.88 (3H, s, H-18), 1.08 (1H, m, H-1α), 1.13 (1H, dd, *J* = 12.6, 3.0 Hz, H-5), 1.22 (1H, m, H-3α), 1.33 (1H, m, H-6β), 1.43 (1H, m, H-3β), 1.52 (1H, m, H-2α), 1.60 (1H, m, H-2β), 1.70 (1H, m, H-1β), 1.76 (1H, m, H-6α), 1.91 (1H, br d, *J* = 10.9 Hz, H-9), 2.02 (1H, dt, *J* = 13.0, 4.9 Hz, H-7α), 2.35 (1H, m, H-11a), 2.57 (1H, ddd, *J* = 13.6, 6.1, 2.8 Hz, H-11b), 2.43 (1H, m, H-7β), 3.33 (1H, AB q, *J* = 16.5 Hz, H-14a), 3.35 (1H, AB q, *J* = 16.5 Hz, H-14b), 4.38 (1H, s, H-17a), 4.85 (1H, s, H-17b), 6.69 (1H, t, *J* = 6.5 Hz, H-12); ^{13}C-NMR (100 MHz, $CDCl_3$): δ 14.42 (C-20), 19.29 (C-2), 21.74 (C-19), 24.11 (C-6), 24.61 (C-11), 29.57 (C-14), 35.59 (C-4, 18), 37.86 (C-7), 39.22 (C-1), 39.59 (C-10), 42.00 (C-3), 55.39 (C-5), 56.39 (C-9), 107.90 (C-17), 135.68 (C-13), 159.42 (C-12), 148.05 (C-8), 174.80 (C-15), 193.68 (C-16); FTIR (neat): ν = 3449, 2929, 1708, 1389, 730 cm^{-1}.

Zerumin B (**10**): colorless crystal; m.p = 108.0–109.0 °C; $[\alpha]_D^{29}$ = +28.77 (*c* 0.51, $CHCl_3$); HREIMS: $[M + Na]^+$ 357.2029 (calcd for $C_{20}H_{30}O_4Na$, 357.2042); ^{1}H-NMR (400 MHz, $CDCl_3$): δ 0.67 (3H, s, H-20), 0.80 (3H, s, H-19), 0.88 (3H, s, H-18), 1.06 (1H, dt, *J* = 12.4, 4.0 Hz, H-1α), 1.17 (1H, m, H-5), 1.22 (1H, m, H-3α), 1.33 (1H, m, H-6β), 1.41 (1H, m, H-3β), 1.50 (1H, m, H-2α), 1.58 (1H, m, H-2β), 1.65 (1H, m, H-11a), 1.69 (1H, m, H-1β), 1.76 (1H, m, H-6α), 1.83 (1H, m, H-11b), 2.04 (1H, m, H-7α), 2.06 (1H, m, H-9), 2.40 (1H, br d, *J* = 12.3 Hz, H-7β), 4.65/4.69 (1H, s, H-17b), 4.87/4.88 (1H, s, H-17a), 4.51 (1H, br d, *J* = 9.7 Hz, H-12), 7.04/7.07 (1H, s, H-14); ^{13}C-NMR (100 MHz, $CDCl_3$): δ 14.49/14.61 (C-20), 19.33 (C-2), 21.69/21.94 (C-19), 24.36 (C-6), 29.87/30.14 (C-11), 33.60 (C-4, 18), 38.24/38.27 (C-7), 38.94 (C-1), 38.80/39.89 (C-10), 42.05 (C-3), 55.43/55.50 (C-5), 51.79/51.90 (C-9), 65.46/66.16 (C-12), 97.57/98.42 (C-15), 106.88/107.66 (C-17), 142.21 (C-13), 144.01/144.10 (C-14), 148.21/148.27 (C-8), 171.05/171.09 (C-16); FTIR (neat): ν = 3417, 2941, 1746, 1642 cm^{-1}.

Table S1. ^{1}H-, ^{13}C-NMR and HMBC data of compounds **6** and **7**.

Positions	δ ^{1}H (Mult., *J* in Hz)			δ ^{13}C		HMBC	
	Compound 6	Compound 7	Ref. (Kiem *et al.*, 2012)	Compound 6	Compound 7	Compound 6	Compound 7
1 [a]	α 0.99 (*dt*, 13.1, 2.6)	α 1.02 (*m*)	1.00 (dt, 3.5, 13.0)	40.82	40.84; 40.99	C-2, 9, 10	C-2, 3, 10, 20
	β 1.47 (*m*)	β 1.38 (*m*)	1.45				
2 [a]	α 1.41 (*m*)	α 1.41 (*m*)	1.42	19.07	19.02; 19.07	C-1, 3	C-1, 3, 4
	β 1.52 (*m*)	β 1.54 (*m*)	1.50				
3	α 1.18 (*dt*, 13.3, 3.5)	α 1.20 (*m*)	1.18 (dt, 3.5, 12.5)	42.24	42.14	C-1, 2, 4, 5	C-1, 2, 4, 18, 19
	β 1.39 (*m*)	β 1.43 (*m*)	1.44				
4	–	–	–	33.57	33.55		
5 [a]	1.08 (*dd*, 12.5, 2.3)	1.09 (*dd*, 12.6, 2.3)	1.09 (dd, 2.5, 12.5)	54.66	54.51; 54.53	C-4, 6, 9, 10, 20	C-4, 6, 10, 20
6	α 1.37 (m)	α 1.40 (*m*)	1.39	23.32	23.22	C-5, 7, 8, 10	C-5, 7, 8, 10
	β 1.71 (m)	β 1.71 (*m*)	1.71				
7 [a]	α 2.07 (*dt*, 13.1, 5.0)	α 2.10 (*m*)	2.08 (dt, 5.0,13.0)	36.71	36.59; 36.62	C-5, 6, 8, 9, 17	C-5, 6, 8, 9, 17
	β 2.43	β 2.44 (*m*)	2.43				
8 [a]	–	–	–	149.18	148.61; 148.90		
9 [a]	2.37 (*br d*, 10.1)	2.47 (*br d*, 10.6)	2.38 (d, 9.5)	62.25; 62.26	62.16; 62.11	C-5, 8, 10, 11, 12, 17, 20	C-8, 10, 11, 12,
10 [a]	–	–	–	39.34	39.50; 39.61		
11 [a]	6.97 (*dd*, 15.5, 10.1);	6.58 (*dd*, 16.0, 10.4)	6.96 (dd, 10.0, 16.0)	139.55	144.05; 144.13	C- 8, 9, 10, 12, 13	C- 8, 9, 10, 13
	6.95 (*dd*, 15.5, 10.7)	6.62 (*dd*, 16.0, 10.4)	2.46 (m)	–	–		
12 [a]	6.08 (*d*, 15.8)	6.31 (*d*, 16.0)	6.10 (d, 16.0)	120.27; 120.29	122.72; 122.78	C-8, 9, 10, 11, 13, 16	C-9, 10, 13, 14, 16
13	–	–	–	132.90	161.52		
14	6.79 (*br s*)	5.85 (*br s*)	6.78 (s)	139.27	115.33	C-12, 13, 15, 16	C-11, 12, 13, 15, 16
15	5.76 (*br s*)		5.76 (s)	101.95	172.03	C-12, 13, 14, 16, 21	
16	–	6.27 (*s*);6.29 (*s*)	–	169.62	98.00; 98.02	C-12, 13, 14	C-14, 15
17 [a] a	4.47 (*br s*)	4.38 (*br s*)/4.48 (*br s*)	4.76 (br s)	108.49; 108.53	108.53; 108.97	C-7, 8, 9	C-7, 8, 9
17 [a] b	4.76 (*br s*)	4.78 (*br s*)	4.48 (*br s*)	–	–		
18	0.89 (*s*)	0.89 (*s*)	0.89 (*s*)	33.57	33.58	C-3, 4, 5, 19	C-3, 4, 5, 19
19	0.84 (*s*)	0.84 (*s*)	0.84 (*s*)	21.93	21.93	C-3, 4, 5, 18	C-3, 4, 5, 18
20 [a]	0.87 (*s*)	0.86 (*s*)	0.87 (*s*)	15.07	15.09; 15.16	C-1, 5, 9, 10	C-1, 5, 9, 10
-OC<u>H</u>$_3$ [a]	3.57/3.58 (*s*)	–	3.56 (s)	56.83; 56.91	–	C-15	

[a] Signals appeared in pair.

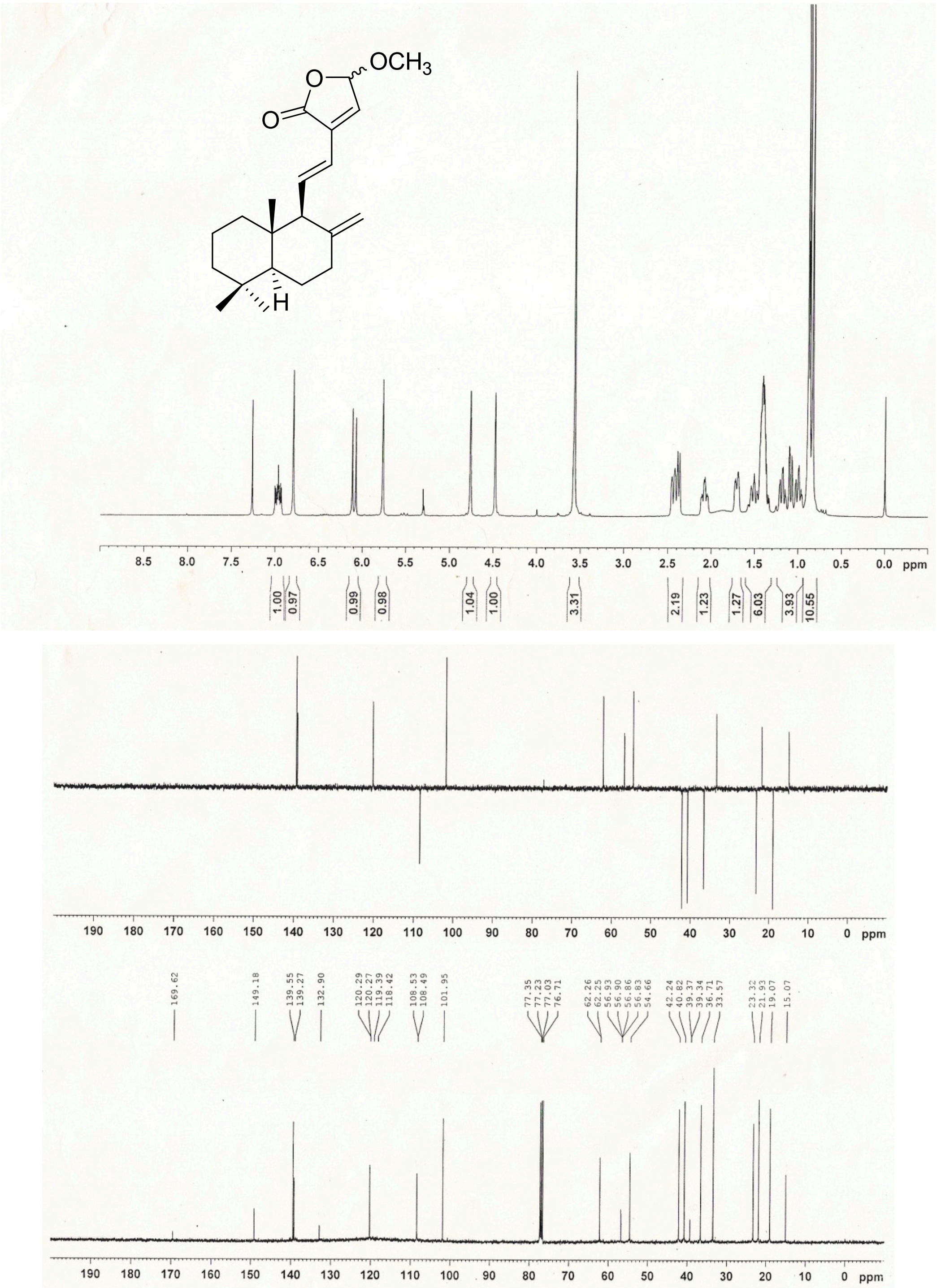

Figure S1. ^{1}H, ^{13}C and DEPT 135 NMR spectra of 15-methoxylabda-8(17),11,13-trien-15,16-olide (**6**) ($CDCl_3$).

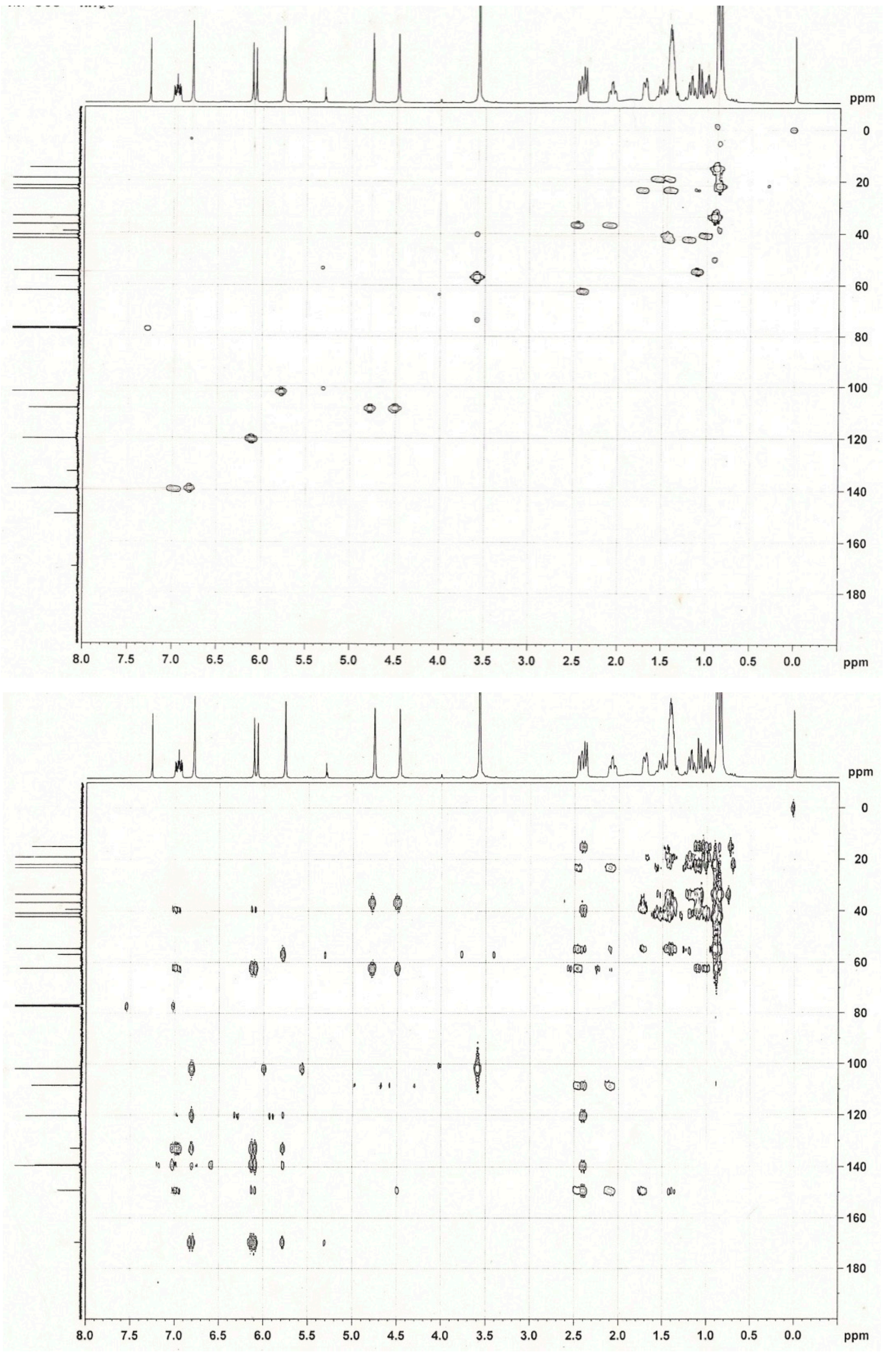

Figure S2. HMQC and HMBC spectra of 15-methoxylabda-8(17),11,13-trien-15,16-olide (**6**).

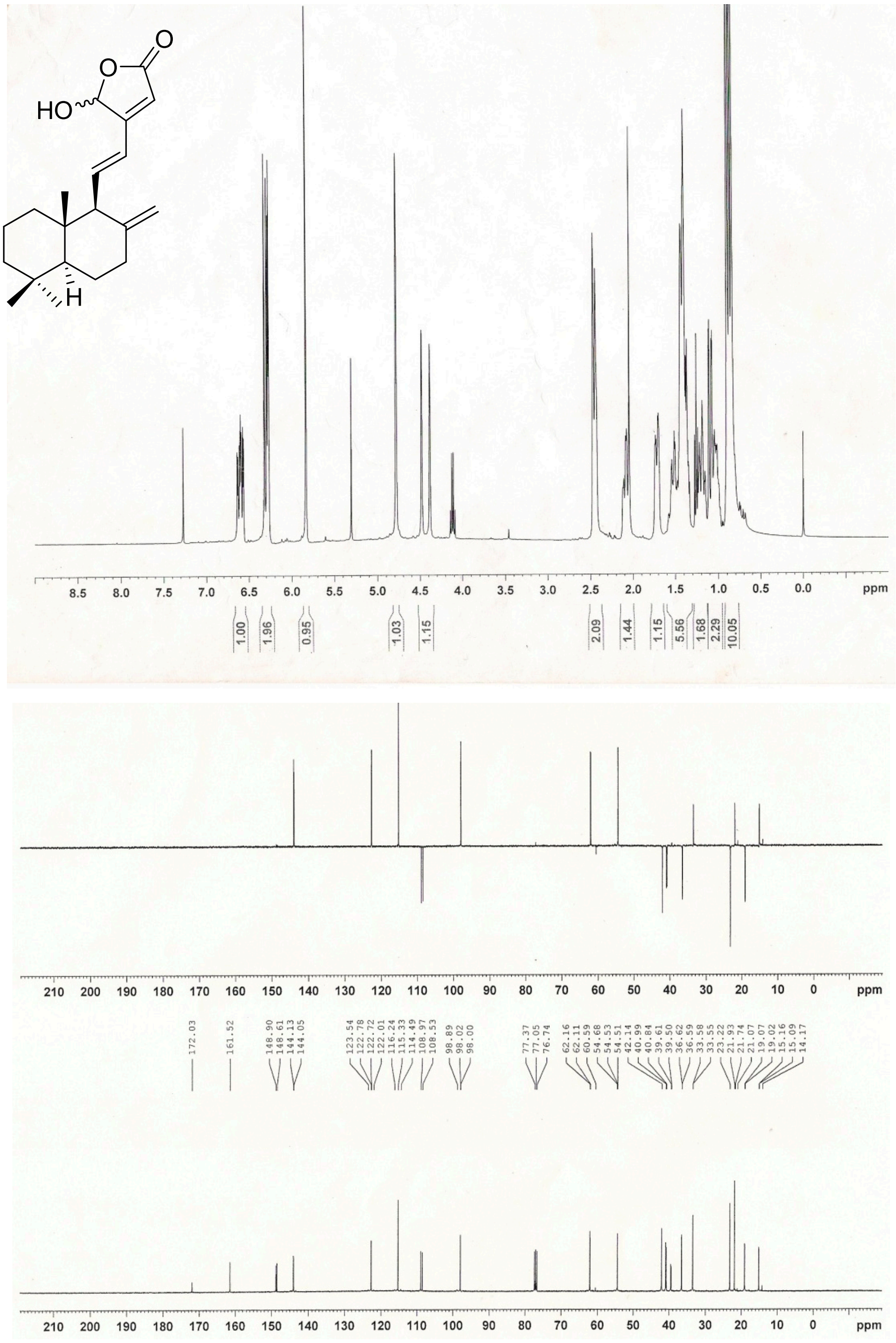

Figure S3. ^{1}H, ^{13}C and DEPT 135 NMR spectra of 16-hydroxylabda-8(17),11,13-trien-15,16-olide (**7**) ($CDCl_3$).

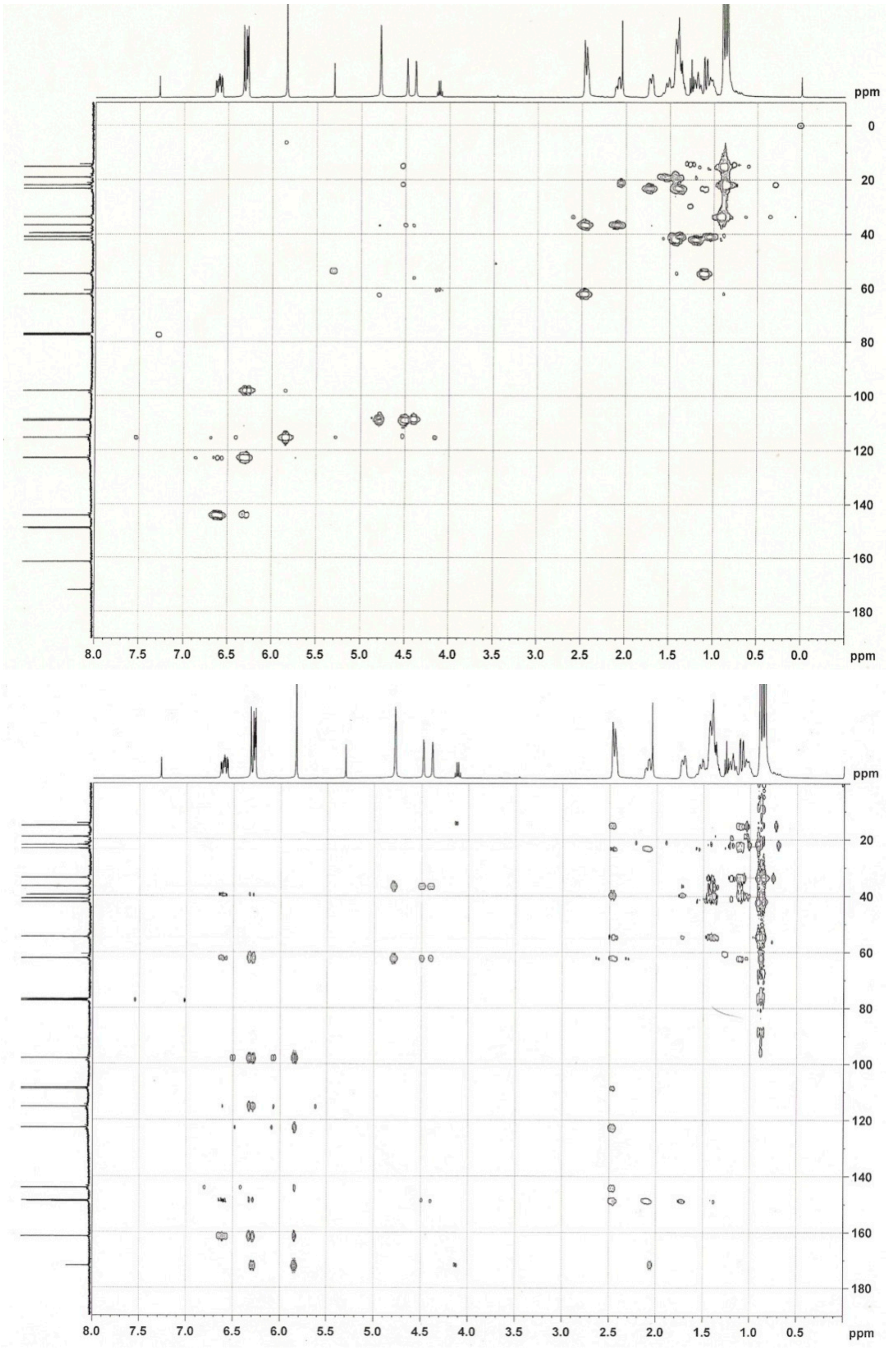

Figure S4. HMQC and HMBC spectra of 16-hydroxylabda-8(17),11,13-trien-15,16-olide (**7**).
